# Erythropoietin in Acute Kidney Injury (EAKI): a pragmatic randomized clinical trial

**DOI:** 10.1186/s12882-022-02727-5

**Published:** 2022-03-13

**Authors:** Mabel Aoun, Ghassan Sleilaty, Celine Boueri, Eliane Younes, Kim Gabriel, Reine-Marie Kahwaji, Najla Hilal, Jenny Hawi, Rita Araman, Dania Chelala, Chadia Beaini

**Affiliations:** 1 Faculty of Medicine, Saint-Joseph University, Beirut, Lebanon; 2 Saint-George Hospital, Ajaltoun, Lebanon; 3 Hopital Sacre-Coeur, Baabda, Lebanon; 4 Serhal Hospital, Rabieh, Lebanon; 5 Middle-East Institute of Health, Bsalim, Lebanon; 6 Bellevue Medical Center, Mansourieh, Lebanon

**Keywords:** Acute kidney injury (AKI), Anemia, Erythropoietin (EPO), Hemoglobin, Death, Dialysis, Randomized pragmatic clinical trial

## Abstract

**Background:**

Treatment with erythropoietin is well established for anemia in chronic kidney disease patients but not well studied in acute kidney injury.

**Methods:**

This is a multicenter, randomized, pragmatic controlled clinical trial. It included 134 hospitalized patients with anemia defined as hemoglobin < 11 g/dL and acute kidney injury defined as an increase of serum creatinine of ≥ 0.3 mg/dL within 48 h or 1.5 times baseline. One arm received recombinant human erythropoietin 4000 UI subcutaneously every other day (intervention; *n* = 67) and the second received standard of care (control; *n* = 67) during the hospitalization until discharge or death. The primary outcome was the need for transfusion; secondary outcomes were death, renal recovery, need for dialysis.

**Results:**

There was no statistically significant difference in transfusion need (RR = 1.05, 95%CI 0.65,1.68; *p* = 0.855), in renal recovery full or partial (RR = 0.96, 95%CI 0.81,1.15; *p* = 0.671), in need for dialysis (RR = 11.00, 95%CI 0.62, 195.08; *p* = 0.102) or in death (RR = 1.43, 95%CI 0.58,3.53; *p* = 0.440) between the erythropoietin and the control group.

**Conclusions:**

Erythropoietin treatment had no impact on transfusions, renal recovery or mortality in acute kidney injury patients with anemia.

The trial was registered on ClinicalTrials.gov (NCT03401710, 17/01/2018).

**Supplementary Information:**

The online version contains supplementary material available at 10.1186/s12882-022-02727-5.

## Background

At the beginning of the 90 s, following the release of the recombinant human erythropoietin (rhuEPO), transfusions' needs to treat anemia in chronic kidney disease patients were minimized [1]. This has been a revolutionary step in the management of renal anemia in chronic kidney disease and led to a major decrease in hepatitis B and C transmissions in dialysis [2]. However, using erythropoietin (EPO) to treat anemia in acute kidney injury (AKI) remains controversial.

AKI is a common disease with a global incidence estimated at 21% and tends to occur more frequently in the critical care setting [3]. The RIFLE and AKIN criteria for AKI definition were merged by the Kidney Disease Improving Global Outcomes (KDIGO) work group. AKI is defined as an increase in serum creatinine (SCr) ≥ 0.3 mg/dL (≥ 26.5 μmol/L) within 48 h or an increase in SCr to ≥ 1.5 times baseline within the last 7 days or a urine volume of < 0.5 mL/kg/h for 6 h [4]. Hales et al. reported that the majority of patients admitted with AKI have anemia (91%) [5]. The presence of anemia in their study was related to the oliguria and uremia level [5]. EPO is secreted by the tubulo-interstitial renal cells and it has been demonstrated that a chronic kidney injury would lead to a decrease in EPO secretion [6]. Some experiments have shown that EPO level usually increases within the first 48 h of AKI then drops progressively [7]. Transfusions will be needed if critically ill patients are hospitalized for a long period of time [8]. Transfusions may lead to sensitization and can hinder future transplantation in patients who reach end-stage renal disease. Therefore, it is essential to prevent transfusions in AKI patients.

We searched the literature for “recombinant human erythropoietin” or "erythropoietin" and “acute kidney disease” “acute renal failure” or or “acute kidney injury”. The search did not reveal any clinical trial assessing rHuEPO use in AKI. Throughout this paper, for a simplification purpose, we will use the term erythropoietin (EPO) instead of the recombinant human erythropoietin (rHuEPO). Based on the literature, some studies assessed the role of EPO before AKI occurrence to prevent kidney injury in cardiac surgery patients and contrast-induced nephropathy with conflicting results [9–14]. Some experimental studies demonstrated a favorable effect of EPO and darbepoetin on the ischemic renal injury in rats [15, 16]. A metaanalysis of 10 randomized controlled trials where the majority of patients received a single dose of EPO concluded that EPO does not prevent AKI or dialysis or death [17]. Two recent clinical trials using high doses of EPO of 40,000 UI weekly following cardiac arrest and traumatic brain injury did not show any prevention of AKI and thus no protective renal effect [18, 19].

The role of EPO after the occurrence of AKI is not well studied. In 2005, a retrospective study showed that EPO treatment in acute renal failure patients did not lower the transfusion requirements, the renal recovery or patient survival [20]. However, it included many limitations such as the low dose of EPO used and the retrospective design [20, 21]. A recent clinical trial of children with hemolytic uremic syndrome showed a decrease in transfusion in patients receiving EPO but it included only 10 children [22]. Therefore, a clinical trial that studies the effect of EPO treatment in AKI patients with anemia is needed.

The primary objective of this randomized clinical trial was to compare the need of red blood cell transfusions in patients with AKI and anemia whether receiving or not rHuEPO. The secondary objectives were to compare the renal survival, the need for dialysis and patient death between the two groups.

## Methods

### Trial design

This was a randomized, controlled, multicenter, pragmatic clinical trial. Patients were randomly assigned in a 1:1 ratio to one of two groups. The intervention group received the EPO treatment and the control group received standard care without EPO. This study aimed to compare the effect of EPO use on transfusion need and other outcomes in acute kidney injury with anemia against no use of EPO.

### Participants and eligibility criteria

Patients included in this trial were admitted to one of the five hospitals where the investigators of this study were practicing: Saint-George Ajaltoun Hospital, Bellevue Medical Center, Serhal Hospital, Sacre-Coeur Hospital, Middle-East Institute of Health.

All adult patients > 18 years old hospitalized with acute kidney injury and anemia were eligible. Acute kidney injury was defined based on the RIFLE, AKIN and KDIGO criteria, as an increase of serum creatinine of ≥ 0.3 mg/dL within 48 h or 1.5 times the baseline level. Anemia was defined in this trial as requiring erythropoietin if Hb < 11 g/dl. Since the decrease in hemoglobin levels can be very rapid in acute settings due mainly to inflammatory causes and since the onset of action of erythropoietin takes several days, we started erythropoietin before the patient reaches lower levels of hemoglobin.

Eligible patients were included after giving their informed consent to participate.

Exclusion criteria were: pregnant women, terminally ill patients, active bleeding, patients with major or minor thalassemia, patients on dialysis and patients who were receiving rHuEPO or any erythropoiesis-stimulating agent (ESA) before admission.

### Data collection

Data for presumed cause of acute kidney injury, comorbidities, medications and laboratory results were collected from the patients' medical records. The following variables were studied: age, gender, home altitude, body mass index (BMI), diabetes, current smoking status, hypertension, hyperlipidemia, previous cardiovascular disease, chronic inflammatory disease, previous chronic obstructive pulmonary disease (COPD), baseline serum creatinine (Scr) if available in the last previous medical record of the patient (with corresponding estimated glomerular filtration rate (eGFR) using the CKD-EPI equation), Scr, hemoglobin level and C-Reactive Protein (CRP) at the time of AKI diagnosis (T1) and before discharge or death (T2). We collected as well serum phosphate, calcium, albumin, bicarbonate, white blood cells, platelets, ferritin, transferrin saturation (TSAT), LDH, vitamin B12, folic acid, reticulocyte count, uric acid and CPK. Data on previous medications intake were collected: iron, non-steroidal anti-inflammatory drugs (NSAIDs), antihypertensive medications specifically renin–angiotensin–aldosterone system inhibitors (RAASi) such as angiotensin-converting enzyme (ACE) inhibitors and angiotensin receptor blockers (ARBs), antiplatelet and anticoagulant agents, urate lowering therapy, antibiotics, immunosuppressive treatment and corticosteroids. Medications administered during the hospitalization were collected particularly vasopressors such as noradrenaline, dopamine, furosemide, antibiotics, anticoagulants, vitamins, enteral or parenteral nutrition and proton-pump inhibitors. Data including the number of units of packed red blood cells transfused during hospitalization, average hospital length of stay (LOS), oligo-anuria at any stage of the AKI, need for dialysis and number of days till renal recovery were collected. Adverse events were also noted such as any thrombotic event.

### Ethical considerations

The study got the approval from the ethics committee of Saint-Joseph University number CE-HDF1115 and is in agreement with the Helsinki Declaration of 1975. The patients signed an informed consent before entering the trial. The trial is registered on ClinicalTrials.gov (NCT03401710, 17/01/2018). Informed consent was signed by the patient or a member of his family. Each participant was assigned two numbers, one for the unit and another for each individual. The data analyst was blinded regarding group allocation.

### Interventions

Patients were randomly assigned to one of two groups: Group 1 received erythropoietin (EPO) 4000 UI every other day subcutaneously (three doses per week) until discharge from the hospital or death and EPO treatment was planned to be stopped if Hb reached 12 g/dl and above. Group 2 received the usual treatment. Treatment was started within 24 h of the diagnosis of concomitant AKI and anemia.

### Outcomes

#### Primary outcome

Need for red blood cell transfusion during the hospitalisation.

#### Secondary outcomes

-Renal recovery whether full recovery defined as a decrease of serum creatinine at discharge to the patient’s baseline or to less than 1.5 mg/dl or partial recovery defined by any reduction in serum creatinine at discharge.

-Need for dialysis.

-All-cause mortality.

### Sample size calculation

We made the assumption that the need for transfusion would be reduced by 40% by the intervention (EPO treatment). If we consider a two-sided alpha of 5% and power of 80% and an effect size = 0.4 (Cohen’s effect size, i.e., standardized mean), the total sample size needed would be 198 patients, 99 patients in each arm.

### Randomization

Patients were assigned to receive EPO or not, using a 1:1 allocation ratio. We used the randomization plan from the www.randomization.com to generate the random allocation sequence. Each time one of the investigators enrolled a new participant, the others were informed. Being a pragmatic trial, the investigators and patients were not blinded to treatment.

#### Pragmatic trial

After randomization, the investigators were free to treat and manage the patient based on their usual real-world practice.

#### Participant timeline

Every patient was followed from first day of acute kidney injury until discharge or transfer or death.

### Statistical analysis

Continuous variables are presented as mean ± standard deviation (SD) if normally distributed and as median and interquartile range (IQR) if skewed. Categorical variables are reported as numbers and percentages. Differences between the two groups of the trial were compared using Chi Square test for categorical variables and Mann–Whitney or t independent test for continuous variables. The risk ratio was calculated for each outcome with the confidence interval (CI). A categorical regression analysis was performed to assess the factors associated with each outcome in the two groups of the trial. Statistical analysis was performed using the Statistical Package for the Social Sciences (IBM SPSS, version 24). A *p*-value of < 0.05 was considered statistically significant.

As of 25/08/2021, a total of 134 patients had been recruited into the study, accounting for 68% of the planned sample size. No further patients could be recruited for several months due to Covid-19 outbreak and the disruption of the usual management of the patients. A decision was reached by the PI, the investigators, the statistician and the ethics committee to terminate prematurely the trial, given the forced zeroing of recruitment rate and the quasi-impossibility of reaching the planned sample size within a reasonable time.

A post-hoc power analysis was performed for the primary outcome for transparency purposes, calculating the power to detect the initially planned effect size of 0.4 with the effective sample size (ref: The 20% Statistician: Observed power, and what to do if your editor asks for post-hoc power analyses (daniellakens.blogspot.com)). Power analysis was performed using GPower software v3.192 (ref: Faul, F., Erdfelder, E., Lang, A.-G., & Buchner, A. (2007). G*Power 3: A flexible statistical power analysis program for the social, behavioral, and biomedical sciences. Behavior Research Methods, 39, 175–191).

## Results

### Participant flow

**Fig. 1:**
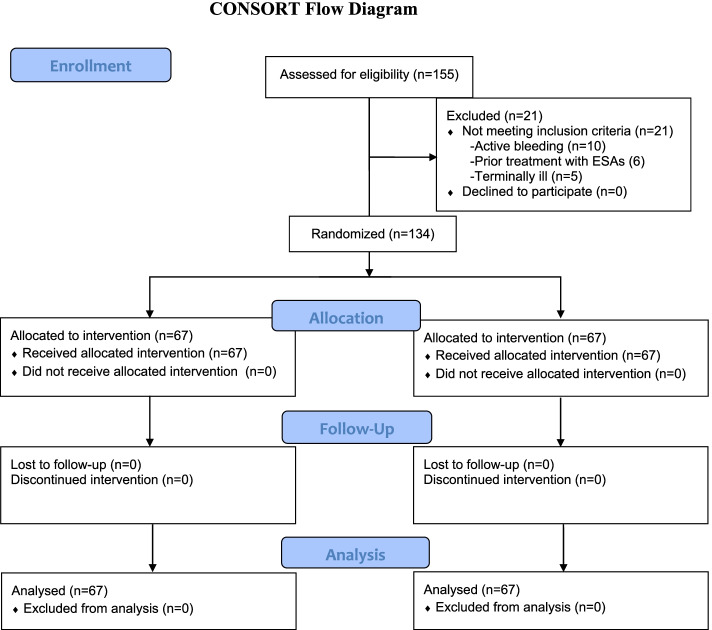
CONSORT flow diagram

One hundred and thirty-four patients were randomly assigned to receive either erythropoietin (*n* = 67) or standard of care (*n* = 67). No patient was lost or excluded after randomization (Fig. 1). All patients in the EPO arm received the treatment until discharge from the hospital or death.

### Recruitment

The trial was terminated before reaching the planned sample of 198 patients because of slow recruitment. During the peak of the coronavirus disease (COVID-19) pandemic, no patients were included.

### Baseline data

Demographics and baseline clinical and biological characteristics of both groups are listed in Table 1. The hemoglobin T2 (after treatment with EPO) did not exceed 12 g/dL in all patients, thus treatment was not stopped in any patient. Seven out of the 134 patients had missing values for a previous serum creatinine or baseline eGFR.

**Table 1:** Characteristics for AKI patients treated with EPO vs no EPO treatment

	**Patients without EPO** ***n* = 67**	**Patients treated with EPO** ***n* = 67**	***p***
**Parameters at baseline T1**			
** Age, mean** ± **SD** ** Median (IQR)**	78.82 ± 12.04 81 (73, 86)	76.42 ± 12.98 79 (69, 86)	0.269^a^
** Gender M/F, n(%)**	34/33 (50.7/49.3)	37/30 (55.2/44.8)	0.604^b^
** BMI, median (IQR)**	27.26 (23.79, 30.79)	26.12 (24.40, 30.66)	0.723^c^
** Diabetes, n(%)**	33 (49.3)	37 (55.2)	0.489^b^
** Smoking status, n(%)**	12 (17.9)	27 (40.3)	0.005^b^
** Hypertension, n(%)**	63 (94.0)	60 (89.6)	0.531^d^
** Hyperlipidemia, n(%)**	44 (65.7)	50 (74.6)	0.257^b^
** Previous CVD, n(%)**	35 (52.2)	40 (59.7)	0.384^b^
** COPD, n(%)**	11 (16.4)	20 (29.9)	0.072^b^
** Chronic inflammation, n(%)**	20 (29.9)	19 (28.4)	0.849^b^
** RAASi intake, n(%)**	28 (41.8)	26 (38.8)	0.723^b^
** Home altitude in meters, mean** ± **SD**	615.44 ± 405.89	623.56 ± 356.98	0.916^a^
** Presumed cause of AKI**			
ATN/Sepsis	18 (26.9)	20 (29.9)	0.066^b^
ATN/Hypovolemia	13 (19.4)	5 (7.5)	
ATN/Nephrotoxic agents	4 (6.0)	2 (3.0)	
Cardiorenal	10 (14.9)	15 (22.4)	
Other	22 (32.8)	25 (37.3)	
** Baseline eGFR (mL/min/1.73m2), mean** ± **SD**	53.86 ± 21.82	50.52 ± 25.75	0.423^a^
** Baseline eGFR < 60 mL/min, n(%)**	41 (61.2)	44 (65.7)	0.591^b^
** Serum creatinine T1 (mg/dL), median (IQR)**	2.3 (1.7, 4.2)	2.6 (2, 4.4)	0.224^c^
AKI stage 1, n(%)	29 (43.3)	34 (50.7)	0.428^b^
AKI stage 2, n(%)	12 (17.9)	15 (22.4)	
AKI stage 3, n(%)	21 (31.3)	16 (23.9)	
Missing baseline creatinine, n(%)	5 (7.5)	2 (3)	
** Hemoglobin T1 (g/dL), median (IQR)**	9.7 (8.8, 10.5)	9.4 (8.4, 10.0)	0.034^c^
** Serum albumin T1 (g/L), median (IQR)**	32.0 (28.8, 36.0)	31.0 (27.0, 35.0)	0.360^c^
** Serum calcium T1 (mg/dL), median (IQR)**	8.8 (8.3, 9.1)	8.8 (7.8, 9.2)	0.554^c^
** Serum phosphate T1 (mg/dL), median (IQR)**	4.2 (3.4, 5.6)	4.6 (3.9, 6.3)	0.077^c^
** Serum bicarbonate T1 (meq/L), median (IQR)**	22.0 (19.0, 25.0)	21.0 (18.0, 24.0)	0.337^c^
** Ferritin (ng/mL), median (IQR)**	181.75 (61.63, 366.25)	247.0 (99.75, 368.27)	0.564^c^
** TSAT (%), median (IQR)**	12.5 (8.0, 24.3)	10.5 (7.3, 17.4)	0.222^c^
** CRP level T1 (mg/L), median (IQR)**	74.0 (12.0, 168.0)	59.85 (20.8, 148.25)	0.752^c^
** Vitamin B12 (pg/mL), median (IQR)**	322.0 (201.5, 809.5)	347.5 (226.0, 756.75)	0.705^c^
** Folic acid (ng/mL), median (IQR)**	10.5 (6.6, 20.0)	7.9 (5.9, 12.3)	0.163^c^
** LDH (U/L), median (IQR)**	186.5 (164.0, 248.0)	214.5 (174.75, 273.5)	0.088^c^
** Vasopressor use, n(%)**	4 (6)	14 (20.9)	0.021^b^
** Corticosteroid use, n(%)**	21 (31.3)	23 (34.3)	0.666^b^
** Iron intake, n(%)**	13 (19.7)	22 (32.8)	0.067^b^
** Anticoagulation, n(%)**	37 (55.2)	41 (61.2)	0.419^b^
** Oligoanuria, n(%)**	11 (16.4)	18 (26.9)	0.142^b^
** Total dose of EPO UI/Kg, median (IQR)**	0	150 (88.5, 220.5)	< 0.001
**Parameters after follow-up T2**			
** Serum creatinine T2, median (IQR)**	1.4 (1.1, 2.5)	1.8 (1.2, 2.91)	0.167^c^
** Hemoglobin T2, median (IQR)**	9.95 (9.1, 10.6)	9.7 (9.0, 10.5)	0.492^c^
** Percentage of increase in hemoglobin level, mean** ± **SD**	2.19 ± 0.01	6.4 ± 0.01	0.092^a^
** CRP level T2, median (IQR)**	30.0 (14.0, 72.0)	24.5 (12.5, 52.75)	0.381^c^
** Length of stay in days, median (IQR)**	8 (6, 13)	9 (6, 14)	0.342^c^
** Number of units of packed red blood cells transfused, median (IQR)**	0 (0,2)	0 (0, 1.5)	0.991^c^

T1, at time of AKI diagnosis; T2, before discharge or death; AKI stages are defined based on the KDIGO: Stage 1: increase in serum creatinine by 1.5 to 1.9 times baseline; Stage 2: 2–2.9 times baseline; Stage 3: 3 times baseline or more than 4 mg/dL or requiring dialysis

^a^t independent test; ^b^Chi-Square; ^c^Mann-Whitney U test; ^d^Fischer's Exact test

### Outcomes

There was no statistically significant difference in transfusion need (RR = 1.05, 95%CI 0.65,1.68; *p* = 0.855), in renal recovery full or partial (RR = 0.96, 95%CI 0.81,1.15; *p* = 0.671), in need for dialysis (RR = 11.00, 95%CI 0.62, 195.08; *p* = 0.102) or in death (RR = 1.43, 95%CI 0.58,3.53; *p* = 0.440) between the EPO and the control group (Table 2).

**Table 2:** Clinical Outcomes for EPO treatment vs no EPO treatment in AKI patients

	**Patients without EPO** ***n* = 67**	**Patients treated with EPO** ***n* = 67**	**Risk Ratio**	**95%Confidence Interval**	***p***
Primary outcome, n(%)					
Transfusions	22 (32.8)	23 (34.3)	1.05	0.65, 1.68	0.855
Secondary outcomes, n(%)					
Renal recovery to baseline, full or partial	54 (80.6)	52 (77.6)	0.96	0.81, 1.15	0.671
Dialysis	0 (0)	5 (7.5)	11.00	0.62, 195.08	0.102
Death	7 (10.4)	10 (14.9)	1.43	0.58, 3.53	0.440

Post-hoc power determination for detecting the initial effect size of 0.4 yielded 1- β = 52%

### Ancillary analyses

A regression analysis of factors associated with the primary (Table 3) and secondary outcomes (Tables 4 and 5) showed hemoglobin at time of diagnosis of AKI (T1) as significantly associated with need for transfusion in both arms. The multivariate analyses of factors associated with the three outcomes are depicted in Tables S1, S2 and S3.

**Table 3:** Factors associated with transfusions in both arms

	**Group without EPO**			**Group with EPO**		
	**OR**	**95%CI**	***p***	**OR**	**95%CI**	***p***
**Age**	1.03	0.98,1.09	0.250	1.01	0.97,1.05	0.599
**Gender** **Ref: Male**	0.73	0.26,2.03	0.545	1.42	0.51,3.96	0.502
**Smoking**	4.00	1.09,14.66	0.036	0.70	0.25,1.99	0.507
**Diabetes**	1.37	0.49,3.82	0.545	1.08	0.39,2.99	0.877
**Previous CVD**	0.88	0.32,2.43	0.798	1.91	0.66,5.54	0.237
**COPD**	2.93	0.78, 0.97	0.111	0.76	0.25,2.33	0.627
**NSAIDs prior use**	1.37	0.21,8.84	0.743	1.91	0.11,32.01	0.653
**Baseline eGFR**	0.99	0.97,1.02	0.722	0.99	0.97,1.01	0.331
**AKI stage 2 or 3** **Ref: AKI stage 1**	1.05	0.38,2.89	0.932	1.58	0.57,4.35	0.380
**Hemoglobin T1**	0.26	0.13,0.51	< 0.001	0.35	0.19,0.65	0.001
**Serum creatinine T1**	1.01	0.84,1.19	0.958	1.21	0.99,1.46	0.058
**CRP T1**	1.00	0.99,1.01	0.444	1.00	0.99,1.01	0.490
**Platelets' count**	1.00	1.00, 1.00	0.476	1.00	1.00, 1.00	0.055
**Ferritin**	1.00	0.99,1.00	0.278	1.04	0.99,1.00	0.198
**Iron intake**	1.32	0.38,4.65	0.662	0.41	0.13,1.31	0.133
**Anticoagulation**	2.24	0.77,6.54	0.140	3.13	0.98,9.97	0.053
**Corticosteroid use**	3.40	1.14,10.15	0.028	1.29	0.45,3.69	0.641
**Vasopressor use**	6.47	0.63,66.38	0.116	0.69	0.19,2.52	0.580

*OR* odds ratio, *95%CI* 95% Confidence Interval

**Table 4:** Factors associated with death in both arms

	**Group without EPO**			**Group with EPO**		
	**OR**	**95%CI**	***p***	**OR**	**95%CI**	***p***
**Age**	1.03	0.95,1.13	0.475	1.04	0.98,1.12	0.220
**Gender** **Ref: Male**	0.75	0.15,3.64	0.721	3.45	0.81,14.74	0.095
**Smoking**	4.17	0.79,21.84	0.091	0.32	0.06,1.64	0.172
**Diabetes**	1.43	0.29,6.92	0.660	2.10	0.49,8.94	0.316
**Previous CVD**	0.66	0.14,3.19	0.601	1.02	0.26,3.99	0.983
**COPD**						
**Baseline eGFR**	0.99	0.96,1.04	0.970	1.01	0.99,1.04	0.361
**AKI stage 2 or 3** **Ref: AKI stage 1**	There was no death in stage 1			3.45	0.81,14.73	0.095
**Hemoglobin T1**	0.53	0.29,0.94	0.030	0.73	0.39,1.35	0.321
**Serum creatinine T1**	1.17	0.96,1.43	0.128	0.93	0.69,1.24	0.631
**CRP T1**	1.00	0.99,1.01	0.545	1.00	0.99,1.01	0.905
**Ferritin**	1.00	0.99,1.005	0.911	1.001	0.99,1.005	0.638
**Serum albumin**	0.91	0.81,1.02	0.113	0.92	0.83,1.02	0.109
**Serum phosphate**	1.77	1.19,2.62	0.005	1.17	0.92,1.48	0.206
**Serum bicarbonate**	0.86	0.74,0.98	0.027	1.05	0.93,1.19	0.425
**Hemoglobin T2**	0.46	0.27,0.78	0.004	0.82	0.51,1.32	0.421
**CRP T2**	1.01	1.00,1.03	0.034	1.02	1.00,1.03	0.019
**Transfusions**	6.32	1.12,35.79	0.037	3.53	0.88,14.12	0.075
**Full or partial renal recovery**	0.13	0.03,0.69	0.017	0.01	0.001,0.12	< 0.001
**Corticosteroid use**	1.71	0.35,8.43	0.511	4.59	1.03,20.53	0.046
**Vasopressor use**	11.00	1.26,95.69	0.030	9.00	2.07,39.14	0.003

*OR* odds ratio, *95%CI* 95% Confidence Interval

**Table 5:** Factors associated with renal recovery in both arms

	**Group without EPO**			**Group with EPO**		
	**OR**	**95%CI**	***p***	**OR**	**95%CI**	***p***
**Age**	1.04	0.99,1.09	0.139	1.02	0.98,1.06	0.426
**Gender** **Ref: Male**	1.72	0.49,5.94	0.389	0.45	0.14,1.46	0.184
**Smoking**	0.40	0.09,1.62	0.199	2.18	0.61,7.76	0.228
**Diabetes**	0.79	0.24,2.68	0.713	0.78	0.24,2.50	0.673
**Hypertension**	4.73	0.59,37.28	0.140	5.93	1.16,30.42	0.033
**Previous CVD**	0.41	0.11,1.50	0.180	0.98	0.31,3.18	0.979
**COPD**	1.13	0.21,5.97	0.890	1.22	0.34,4.43	0.760
**Baseline eGFR**	1.00	0.98,1.03	0.821	0.99	0.97,1.01	0.464
**Hemoglobin T1**	1.19	0.75,1.89	0.470	1.56	0.91,2.68	0.109
**AKI stage 2 or 3** **Ref: AKI stage 1**	1.72	0.49,5.94	0.389	0.45	0.14,1.46	0.184
**Serum creatinine T1**	0.96	0.79,1.15	0.641	0.94	0.77,1.16	0.579
**Oligoanuria**	0.34	0.08,1.39	0.131	0.06	0.01,0.23	< 0.001
**CRP T1**	1.01	1.001,1.02	0.039	1.01	0.99,1.02	0.134
**Serum phosphate**	0.79	0.59,1.06	0.120	0.78	0.62,0.99	0.044
**Ferritin**	1.00	0.99,1.002	0.775	0.99	0.99,1.003	0.666
**Hemoglobin T2**	0.99	0.61,1.59	0.965	2.48	1.24,4.96	0.010
**CRP T2**	0.99	0.98,1.008	0.701	0.99	0.98,1.008	0.505
**Iron intake**	1.28	0.24,6.70	0.771	3.87	0.78,19.15	0.097
**Corticosteroid use**	1.71	0.42,7.02	0.454	0.46	0.14,1.52	0.202
**Vasopressor use**	0.22	0.03,1.77	0.156	0.28	0.08,1.003	0.051

*OR* odds ratio, *95%CI* 95% Confidence Interval

### Harms

No side-effects of the treatment were noted, specifically thromboembolic events, during the hospitalization of patients.

## Discussion

This is the first randomized clinical trial that assesses the role of erythropoietin treatment in hospitalized patients with acute kidney injury and anemia. It showed no benefit towards the transfusion need or renal recovery or patient survival. This is aligned with the results of the retrospective study of Park et al. in 2005 that evaluated 187 patients including 71 patients who were administered erythropoietin three times weekly at a mean dose of 112 U/kg/week [20]. It was assumed that the retrospective design of that study and the low dose of erythropoietin prevented from showing any positive effect of erythropoietin on reducing transfusions in acute kidney injury patients. However, in our trial, erythropoietin was given at a median dose of 150 UI/kg for a median of 8–9 days and still was not beneficial. It remains unknown whether much higher doses can protect AKI patients from being transfused. A recent study on mice showed a U-shaped effect of EPO receptors on renal prognosis following acute kidney injury [23]. A high dose of 40,000 UI of EPO administered weekly was studied by Corwin et al. in 2002 and 2007 in critically ill patients admitted with anemia [24, 25]. Their patients had no AKI on admission but the goal of their study was to assess the possible reduction of transfusions with EPO [25]. In 2002, they found that patients treated with EPO had less need for transfusions but they had the same mortality rate as the control group [24]. In 2007, there was no significant difference in the transfusions between the group treated with EPO and the placebo [25]. Corwin et al. attributed this difference in the outcomes of the two trials to the TRICC trial that changed the practice in critical care units and decreased the transfusions' requirements until very low hemoglobin levels [25]. However, when Corwin et al. divided their sample to those admitted for trauma, who were in their 40 s, compared to those admitted for medical and surgical reasons, who were in their 60 s, EPO treatment was beneficial in the trauma group [25]. Our study included elderly patients in their 70 s and our results concur well with the elderly group of Corwin et al.

Another question that is worth studying in the future is whether concomitant iron administration could help maintain the hemoglobin level at acceptable ranges. Our patients had features of functional iron deficiency. In chronic kidney disease patients, functional iron deficiency is defined as TSAT levels < 20% and ferritin levels higher than 100 ng/mL [26]. These patients might benefit from intravenous iron. This was not included in the intervention of our trial and iron administration was left to the best practice of each physician, which led only to 20 to 33% of patients taking iron. In the trials of Corwin et al., 100% of patients received oral or parenteral iron over 29 days and this might explain the higher increase of hemoglobin in their patients [25].

An interesting finding in our study is the association of low hemoglobin levels at time of AKI diagnosis with the death outcome. The need for transfusion was also associated with the mortality. This association was statistically significant in patients who did not receive erythropoietin. Anemia has been associated with mortality in chronic kidney disease patients [27]. The combination of anemia and acute kidney injury was demonstrated as associated with increased mortality in several populations such as patients undergoing coronary artery bypass grafting surgery or patients with chronic heart failure [28, 29]. However, this association is not consistent in all studies and a retrospective cohort of 211 patients in 2013 did not find anemia as affecting the renal or the patient survival [30].

The C-Reactive protein level before death or discharge, the lack of renal recovery and the use of vasopressors were the most significantly associated factors with mortality in our AKI patients. Indeed, a lack of response to treatment, persistent inflammatory markers and hemodynamic instability in septic patients with AKI are the most prominent prognostic factors. This is in alignment with previous studies from different countries [31, 32]. A systematic review in 2013 of 154 large studies that evaluated the world incidence of AKI and associated mortality demonstrated a pooled AKI-associated mortality rates of 23.9% and that rate was declining over time [33]. The low rate of mortality in our group of patients not exceeding 15% can be due to the early diagnosis and management of AKI in this clinical trial setting.

Several lessons were learned during this trial. First, anemia is not as prevalent in acute kidney disease patients as previously reported [5]. It is true that we assessed 150 patients with AKI and anemia for eligibility but to reach this step of the process, we had to look at all AKI patients whether they had a hemoglobin less than 11 g/dL. We did not record the exact number of AKI patients admitted but we estimate that half of them did not have a low hemoglobin. Thus, the recruitment for this trial was very slow. This should be considered in future trials to include a higher number of centers to reach a higher number of patients. Second, we included in our study patients with AKI of all etiologies. Patients with pre-renal causes got in remission very quickly and this group of patients should probably not be included in future trials that are assessing erythropoietin treatment which effect takes time. Third, iron supplementation in AKI patients with anemia needs to be further studied whether with or without EPO taking into consideration the high rate of functional iron deficiency in this category of patients. Finally, as long as EPO trials in AKI or critical care setting are not showing benefits on mortality and as long as higher doses might cause thromboembolic complications, the end-point of these trials should focus more on patient-reported outcomes as discussed by Drüeke T. regarding ESA use in chronic kidney disease patients [34].

### Limitations

Our trial has several strengths and limitations. It is the first clinical trial to study the effect of erythropoietin in patients with anemia and acute kidney injury. It is a multicenter randomized trial and the pragmatic design reflects better the effect of this treatment in the real-world practice [35].

The major limitation is the power and the recruitment of 68% of the desired sample.

In conclusion, this pragmatic trial showed that erythropoietin treatment had no impact on transfusions' need, renal recovery or mortality in acute kidney injury patients with anemia.

## Supplementary Information

**Additional file 1. **

**Additional file 2. **

**Additional file 3. **

## Data Availability

All data generated or analysed during this study are included in this published article [and its supplementary information files].

## Abbreviations

rhuEPO: Recombinant human erythropoietin
EPO: Eyrthropoietin
AKI: Acute kidney injury
KDIGO: Kidney Disease Improving Global Outcomes
eGFR: Estimated glomerular filtration rate
CRP: C-reactive protein
TSAT: Transferrin saturation
COPD: Chronic obstructive pulmonary disease
CVD: Cardiovascular disease
RAASi: Renin–angiotensin–aldosterone system inhibitors
ACE: Angiotensin-converting enzyme
ARBs: Angiotensin receptor blockers
LOS: Length of stay
