# Supplementary material for: Erythropoietin in Acute Kidney Injury (EAKI): a pragmatic randomized clinical trial
Source: BMC Nephrol. 2022 Mar 13;23:100. doi: 10.1186/s12882-022-02727-5 (PMC8917943; doi:10.1186/s12882-022-02727-5)
Supplement: Supplementary file 1 — Additional file 1. [file 12882_2022_2727_MOESM1_ESM.docx]

Table S1. Multivariate analysis of factors associated with transfusions in both arms.

|  | **Group without EPO** | | | **Group with EPO** | | |
| --- | --- | --- | --- | --- | --- | --- |
|  | **OR** | **95%CI** | ***p*** | **OR** | **95%CI** | ***p*** |
| **Age** | 1.07 | 0.96, 1.19 | 0.236 | 1.07 | 0.99, 1.14 | 0.067 |
| **Gender**  **Ref: Male** | 0.15 | 0.01, 2.14 | 0.163 | 1.42 | 0.26, 7.79 | 0.688 |
| **Smoking** | 5.24 | 0.56, 49.34 | 0.147 | 0.41 | 0.07, 2.28 | 0.306 |
| **Hemoglobin T1** | 0.11 | 0.03, 0.46 | 0.003 | 0.23 | 0.10, 0.54 | 0.001 |
| **Serum creatinine T1** | 0.94 | 0.71, 1.25 | 0.678 | 1.23 | 0.88, 1.71 | 0.225 |
| **Platelets' count** | 1.00 | 1.00, 1.00 | 0.310 | 1.00 | 1.00, 1.00 | 0.331 |
| **Anticoagulation** | 3.62 | 0.44, 29.86 | 0.231 | 3.59 | 0.64, 20.23 | 0.146 |
| **Corticosteroid use** | 14.52 | 1.72, 122.64 | 0.014 | 0.88 | 0.19, 3.93 | 0.869 |

Note. We included in the multivariate analysis, along with age and gender, variables with *p*<0.1 in the univariate analysis (at least in one arm).
