# Supplementary material for: Erythropoietin in Acute Kidney Injury (EAKI): a pragmatic randomized clinical trial
Source: BMC Nephrol. 2022 Mar 13;23:100. doi: 10.1186/s12882-022-02727-5 (PMC8917943; doi:10.1186/s12882-022-02727-5)
Supplement: Supplementary file 2 — Additional file 2. [file 12882_2022_2727_MOESM2_ESM.docx]

Table S2. Multivariate analysis of factors associated with death in both arms.

|  | **Group without EPO** | | | **Group with EPO** | | |
| --- | --- | --- | --- | --- | --- | --- |
|  | **OR** | **95%CI** | ***p*** | **OR** | **95%CI** | ***p*** |
| **CRP T2** | 1.02 | 0.99,1.04 | 0.098 | 1.03 | 0.99,1.07 | 0.139 |
| **Transfusions** | 4.91 | 0.64,37.75 | 0.127 | 4.68 | 0.22,100.34 | 0.324 |
| **Full or partial renal recovery**  **Ref: no recovery** | 0.11 | 0.01,0.97 | 0.047 | 0.002 | 0.00,0.36 | 0.018 |
| **Vasopressor use** | 2.57 | 0.09,77.32 | 0.587 | 13.29 | 0.75,233.94 | 0.077 |

Note. We included in this model variables that were statistically significant (p<0.05) in the univariate analysis (in both arms). It was impossible to include the high number of variables that had at least in one arm a p<0.1.
