# Supplementary material for: Erythropoietin in Acute Kidney Injury (EAKI): a pragmatic randomized clinical trial
Source: BMC Nephrol. 2022 Mar 13;23:100. doi: 10.1186/s12882-022-02727-5 (PMC8917943; doi:10.1186/s12882-022-02727-5)
Supplement: Supplementary file 3 — Additional file 3. [file 12882_2022_2727_MOESM3_ESM.docx]

Table S3. Multivariate analysis of factors associated with renal recovery in both arms.

|  | **Group without EPO** | | | **Group with EPO** | | |
| --- | --- | --- | --- | --- | --- | --- |
|  | **OR** | **95%CI** | ***p*** | **OR** | **95%CI** | ***p*** |
| **Age** | 1.03 | 0.97,1.09 | 0.383 | 1.06 | 0.96,1.17 | 0.290 |
| **Gender** | 0.83 | 0.16,4.24 | 0.821 | 5.59 | 0.52,60.32 | 0.156 |
| **Hypertension** | 6.57 | 0.41,106.39 | 0.185 | 4.44 | 0.25,79.16 | 0.311 |
| **Oligoanuria** | 1.21 | 0.05,28.44 | 0.906 | 0.03 | 0.01,0.53 | 0.017 |
| **CRP T1** | 1.02 | 1.01,1.04 | 0.024 | 1.01 | 0.99,1.02 | 0.089 |
| **Serum phosphate** | 0.77 | 0.39,1.49 | 0.436 | 1.24 | 0.78,1.97 | 0.356 |
| **Hemoglobin T2** | 1.13 | 0.58,2.20 | 0.729 | 3.77 | 1.29,10.97 | 0.015 |
| **Iron intake** | 0.92 | 0.12,6.92 | 0.934 | 9.41 | 0.66,134.30 | 0.098 |
| **Vasopressor use** | 0.13 | 0.01,6.51 | 0.303 | 0.66 | 0.05,9.28 | 0.760 |

Note. We included in this model, along with age and gender, variables with *p*<0.1 in the univariate analysis (at least in one arm).
